# Clinical features and genetic analysis of two siblings with startle disease in an Italian family: a case report

**DOI:** 10.1186/s12881-019-0779-x

**Published:** 2019-03-12

**Authors:** Teresa Sprovieri, Carmine Ungaro, Serena Sivo, Michela Quintiliani, Ilaria Contaldo, Chiara Veredice, Luigi Citrigno, Maria Muglia, Francesca Cavalcanti, Sebastiano Cavallaro, Eugenio Mercuri, Domenica Battaglia

**Affiliations:** 1 Institute of Neurological Sciences, National Research Council, Loc. Burga, 87050 Mangone, CS Italy; 2 Child Neurology and Psychiatry, Fondazione Policlinico Universitario “A. Gemelli” IRCCS, Rome, Italy; 3 Università Cattolica del Sacro Cuore, Rome, Italy

**Keywords:** Hyperekplexia, Startle disease, GLRA1, NGS

## Abstract

**Background:**

Hyperekplexia also known as Startle disease is a rare neuromotor hereditary disorder characterized by exaggerated startle responses to unexpected auditory, tactile, and visual stimuli and generalized muscle stiffness, which both gradually subside during the first months of life. Although the diagnosis of Hyperekplexia is based on clinical findings, pathogenic variants in five genes have been reported to cause Hyperekplexia, of which *GLRA1* accounts for about 80% of cases. Dominant and recessive mutations have been identified in *GLRA1* gene as pathogenic variants in many individuals with the familial form of Hyperekplexia and occasionally in simplex cases.

**Case presentation:**

In the present study, we describe clinical and genetic features of two Italian siblings, one with the major and one with the minor form of the disease. DNA samples from the probands and their parents were performed by NGS approach and validated by Sanger sequencing. The analysis of the *GLRA1* gene revealed, in both probands, compound heterozygous mutations: c.895C > T or p.R299X inherited from the mother and c.587C > A or p.D98E inherited from the father.

**Conclusions:**

Until now, these two identified mutations in *GLRA1* have not been reported before as compound mutations. What clearly emerges within our study is the clinical heterogeneity in the same family. In fact, even though in the same pedigree, the affected mother showed only mild startle responses to unexpected noise stimuli, which might be explained by variable expressivity, while the father, showed no clear signs of symptomatology, which might be explained by non-penetrance. Finally, the two brothers have different form of the disease, even if the compound heterozygous mutations in *GLRA1* are the same, showing that the same mutation in *GLRA1* could have different phenotypic expressions and suggesting an underling mechanism of variable expressivity.

## Background

Hyperekplexia (HPX) also known as Startle disease (OMIM 149400) is a rare neuromotor hereditary disorder characterized by exaggerated startle responses to unexpected auditory, tactile, and visual stimuli and generalized muscle stiffness, which both gradually subside during the first months of life [1–3]. Exaggerated head-retraction reflex (HRR) consisting of extension of the head followed by violent flexor spasms of limbs and neck muscles elicited by tapping the tip of the nose is observed in most children [4]. Usually intellect is normal but mild cognitive delay may occur [5]. Affected individuals can be successfully treated with clonazepam (CZP), an agonist of the γ-aminobutyric acid type A (GABA-A) [6]. Mutations in the gene encoding the α1 subunit of inhibitory glycine receptor (*GLRA1*, OMIM 138491) mapping to chromosome 5p33.35 were first reported in 1993 to cause autosomal dominant familial hyperekplexia [7]. This α1 subunit contains an extracellular domain (ECD) and a transmembrane domain (TMD) that comprises 4 α-helices, termed TM1-TM4. To date, *GLRA1* mutations have been reported as dominant missense (23%), recessive missense (39%) and recessive nonsense (38%) [8]. In addition, mutations in 4 other genes have been reported to cause HPX; they encode both pre- and postsynaptic proteins involved in glycinergic neurotransmission: *SLC6A5* (glycine transporter 2, solute carrier family 6, member 5), *GLRB* (glycine receptor, beta), *GPHN* (gephyrin), and *ARHGEF9* (Rho guanine nucleotide exchange factor 9) gene [9]. In the present study, we describe clinical and genetic features of two Italian siblings with the same compound heterozygous mutations in *GLRA1* gene but discordant phenotypes.

## Case presentation

**Fig. 1:**
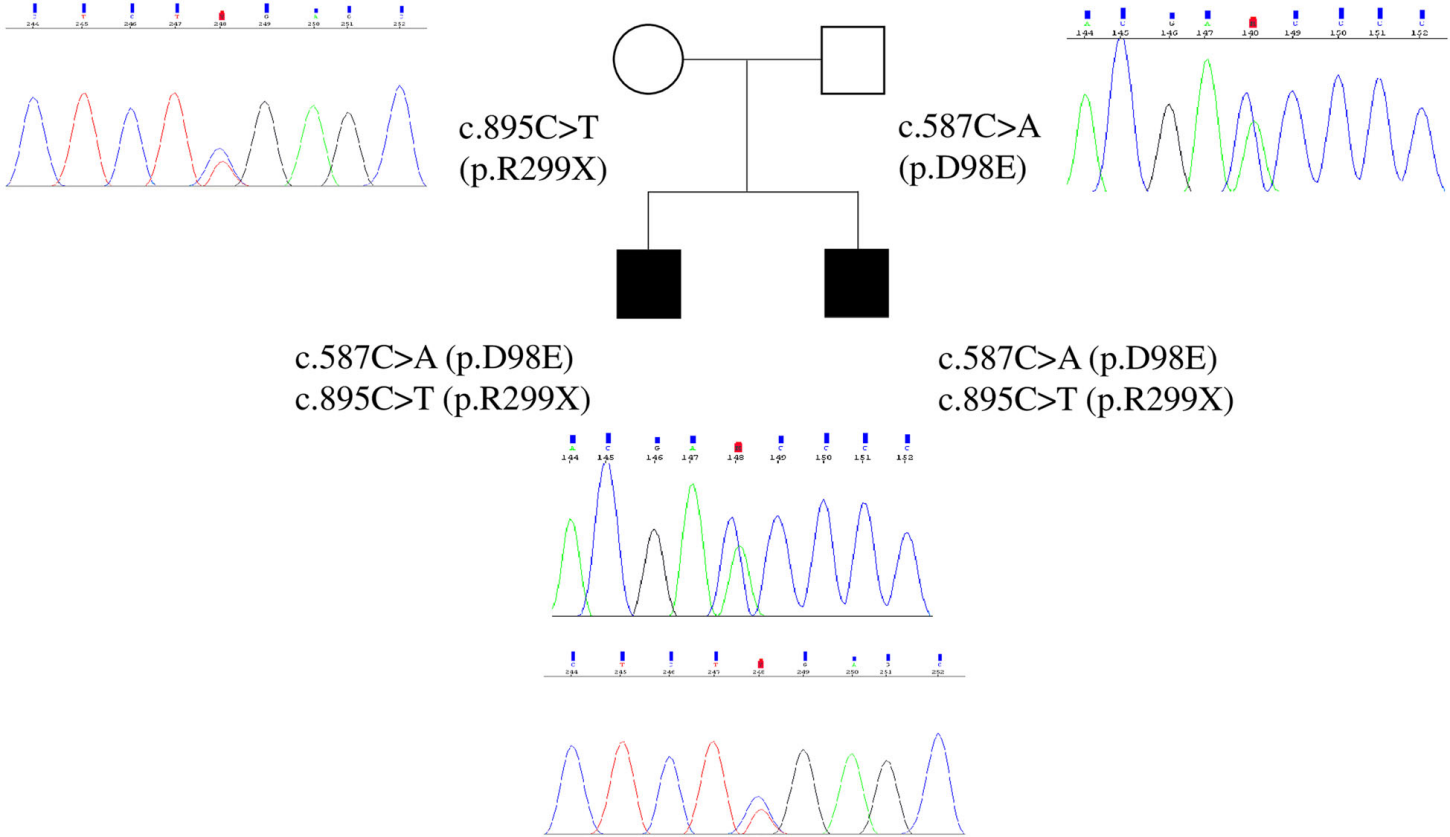
The family pedigree showing the mutations detected in GLRA1

The pedigree for the family is presented in the Fig. 1. Our patients were two siblings, coming from the center of Italy. The younger proband was born at term, from an uneventful pregnancy. At birth he presented with limbs hypertonia and hyperCKemia. During the neonatal period he developed apparently spontaneous episodes, characterized by generalized severe hypertonia with cyanosis, lasting several minutes and occurring during sleep. At the age of two, the child was hospitalized in the local hospital with a diagnosis of epilepsy; no abnormalities were found on both awake and sleep electroencephalogram (EEG) or on brain ultrasounds scan. Phenobarbital treatment was prescribed, but never administered due to parental concerns. The child was referred to our Center, where awake and sleep EEG and cerebral MRI were performed, with normal results. A detailed family history revealed very mild startles in the mother, elicited by sudden noise, and frequent falling episodes in the older brother, causing a reduction of his motor performances. These data were compatible with a possible diagnosis of Hyperekplexia. The nose-tapping test and sudden noise test produced no significant results in the proband but a subsequent episode of generalized hypertonia was triggered by cold gel on the scalp during EEG. This episode was promptly arrested with the Vigevano maneuver (forced flexion of the head and legs towards the trunk) [10]. During the episode the EEG showed normal activities, further suggesting a clinical diagnosis of a neonatal form of Hyperekplexia. O_2_ saturation monitoring during sleep and clonazepam treatment were started, with complete resolution of the episodes. The child developed speech delay in early childhood, and mild behavior disorders (social withdrawal) and no motor impairment. We followed-up the patient until he was 17, no more episodes of hypertonia occurred but he reported occasionally startle episodes triggered by sudden acoustic stimulation, vertigo and trembling hands. An improvement in verbal skills was observed after speech therapy. At the age of 17 his neurological exam, social activity and school abilities are normal. CZP treatment is still ongoing. In consideration of the clinical diagnosis in the younger brother and because of the reported episodes, the older proband was examined at our Center, when he was 4 year old. He was born at term, from uneventful pregnancy and had normal neuromotor and cognitive development. Since the age of 2 years, he had been presenting several episodes of limbs hypertonia, associated with loud crying, during sleep and triggered by sudden noise. These episodes slowly decreased over the years, but the child showed episodes of sudden falling and generalized hypertonia that affected his motor performances and social activities. CZP treatment was started with significant clinical improvement. At the age of 10 an attempt to reduce drug dose caused a relapse of his usual startle episodes trigged by sudden noise. All the EEG performed in both patients were normal, except for high frequency rhythm on the anterior regions, due to benzodiazepine treatment. The mother reported mild startle triggered by sudden noise, without impairment in daily activities, so no therapy was prescribed. The father was completely asymptomatic and in good general condition.

Blood samples were collected from the family members, after informed consent was obtained from all of them. Genomic DNA was isolated from peripheral blood leukocytes using the salting out method. Both probands were screened using an amplicon-based gene panel that included 750 OMIM genes (including *GLRA1*, *GLRB*, *SLCA*, *GPHN*, and *ARHGEF9*) associated with neurological disorders. Genes were sequenced on an Ion Torrent™ Personal Genome Machine™ (PGM) sequencer (ThermoFisher Scientific) with a PCR amplicon-based library preparation (AmpliSeq™) using the ProFlex PCR System (ThermoFisher Scientific) and the Ion AmpliSeq™ Library Kit 2.0 reagents. The amplified library was quantified with the Invitrogen™ Qubit™ Fluorometer and the dilution factor resulting in a concentration of ~ 100 pM was determined. The amplified libraries was enriched using the Ion PGM™ Hi-Q™ View OT2 Kit, then purified with the Ion OneTouch™ ES before loading on a 316-chip and sequenced with an Ion PGM™ Hi-Q™ View Sequencing Kit reagents. After the sequencing run, the bioinformatic analysis, such as quality and coverage analysis, alignment against the GRCh37/hg19 human reference genome (Genome Reference Consortium Human Build 37, https://www.ncbi.nlm.nih.gov/assembly/GCF_000001405.13/) and variant calling were performed using the Torrent Suite™ Software (ThermoFisher Scientific) with the Ion Torrent Server (Dell™). The obtained Variant Caller Format (VCF) files were annotated using wANNOVAR tool (http://wannovar.wglab.org/) and compared against several databases such as the ExAC (Exome Aggregation Consortium, http://exac.broadinstitute.org/), 1000 Genomes (IGSR: The International Genome Sample Resource, http://www.internationalgenome.org/), gnomAD (Genome Aggregation Database, https://gnomad.broadinstitute.org/). The potential functional role of the revealed mutations was predicted using PolyPhen-2 (Polymorphism Phenotyping v2, http://genetics.bwh.harvard.edu/) and SIFT (Sorting Intolerant From Tolerant, https://sift.bii.a-star.edu.sg/). In order to validate the *GLRA1* variations highlighted by NGS approach and in order to perform segregation analysis, DNA samples from the probands and their parents were screened using Sanger sequencing: exons 4 and 7 out of nine and exon-intron boundaries of *GLRA1* gene (ref. seq.: NM_000171.3) were amplified by polymerase chain reaction using sets of oligonucleotide primers specific for exons 4 and 7 and a thermal cycler (Applied Biosystems, Foster City, CA, USA). Primer sequences and PCR conditions are available on request. PCR products were purified and directly sequenced in both forward and reverse directions on an ABI Prism 3130XL genetic analyzer (Applied Biosystems, Foster City, CA) using the BigDye Terminator Cycle Sequencing Ready Reaction Kit (Applied Biosystems).

Molecular analysis of the *GLRA1* gene revealed, in both parents and probands, previously described mutations as reported below: 1) the mother had the heterozygous c.895C > T or p.R299X (rs757488419), located in TM3 domain; 2) the father carried the heterozygous c.587C > A or p.D98E (rs199639315), located in ECD; 3) both probands showed these mutations in a compound heterozygous state, p.R299X inherited from the mother and p.D98E inherited from the father. Molecular variants identified are reported in Fig. 1. The p.R299X variation, first described by Lee et al. in a heterozygous state [9], showing an ExAC MAF = 0,000008/1, was a C-to-T substitution at the nucleotide position 895 in exon 7, replacing Arginine to a premature stop codon at codon 299. The other c.587C > A, showing a frequency T = 0.00001 (1/125568, TOPMED, https://www.ncbi.nlm.nih.gov/snp/rs199639315), was a heterozygous missense mutation in exon 4. Both PolyPhen-2 (score = 0,769, possibly damaging) and SIFT (score = 0.03, deleterious) analysis showed that p.D98E mutation affected negatively gene function. No mutations were found in other genes known to cause familial hyperekplexia such as *GLRB*, *SLC6A5*, *GPHN*, and *ARHGEF9*.

## Discussion and conclusions

Hyperekplexia, or Startle disease, is an uncommon non-epileptic disorder, classically characterized by exaggerated startle responses to unexpected stimuli. The abnormal startle response triggered by nose tapping is pathognomonic in hyperekplexia patients and is generally included in the examination of patients with suggestive features. In patients with hyperekplexia, no abnormalities are observed on routine blood tests, urinalysis, brain imaging studies, or EEG. Because of the overlapping clinical signs, hyperekplexia can be misdiagnosed as epilepsy, cerebral palsy, anxiety disorder, or conversion disorder [11]. Correct diagnosis is however important as it is potentially treatable [12]. Although most antiepileptic drugs are ineffective, benzodiazepines, in particular CZP, can relieve symptoms by the first 2 months of life, if not in the neonatal period [13]. In 1966, Suhren and colleagues [14] described two clinical forms of the disorder: a major and a minor form. The major form of hyperekplexia is characterized by generalized stiffness after birth, normalizing during the first years of life, and excessive startling to an unexpected stimulus, particularly auditory stimuli, that lasts throughout life.

The minor form of hyperekplexia only has excessive startle reflexes. In 2006, Bakker et al. confirmed the presence of two forms of hyperekplexia, identifying a difference in the onset, consisting in the fact that in minor form of hyperekplexia startles never begin in the neonatal period. Both forms can occur in the same pedigree [6]. So far, however in the reported cases in which both major and minor forms occurred in the same pedigree, no mutations of *GLRA1* had been identified. Although the diagnosis of HPX is based on clinical findings, pathogenic variants in five genes have been reported to cause HPX, of which *GLRA1* accounts for about 80% of cases. In fact, *GLRA1* gene encoding the α1 subunit of the glycine receptor, is the major genetic cause of HPX. Dominant missense and recessive missense mutations have been identified as pathogenic variants in many individuals with the familial form of HPX and occasionally in simplex cases [6, 16, 17]. In particular, dominant mutations, located in and around the TM2 domain, do not impair cell surface expression, but disrupt the receptor function by either inducing spontaneous channel activity or by reducing glycine sensitivity, chloride conductance and/or open probability, resulting in a partial loss of function. In contrast, recessive and compound heterozygous mutations mainly affected cell surface trafficking and insertion of receptors into the membrane [18]. The *GLRA1* coding region is distributed over nine exons. Hyperekplexia mutations, clustering in exons 7 and 8, induce amino acid substitutions within a region ranging from TM1 to the extracellular loop connecting segments TM2 and TM3 [19]. To date, no specific genotype-phenotype correlations are known in HPX [4]. Our patients had compound heterozygous mutations: c.895C > T (p.R299X) and c.587C > A (p.D98E). The p.R299X, leading to a premature stop codon, could suppress normal *GLRA1* channel function; p.D98E, in accordance with previously studies reported in literature [20], could impair expression at the cell membrane, requiring much higher glycine levels for channel activation. Patients’mother had the c.895C > T (p.R299X) mutation, and their father had the c.587C > A (p.D98E) mutation. Until now, these two identified mutations in *GLRA1* have not been reported before as compound mutations. Overall, the penetrance of HPX is 100%; however, Kwok et al. (2001), described in one family a mother who had the same variant as her two affected children and was asymptomatic [21]. What clearly emerges within our study is the clinical heterogeneity in the same family. In fact, even though in the same pedigree, the affected mother, carrying the c.895C > T mutation, showed only mild startle responses to unexpected noise stimuli, which might be explained by variable expressivity. The father, carrying the c.587C > A, showed no clear signs of symptomatology, which might be explained by non-penetrance; in fact non-penetrance of mutations in the *GLRA1* gene has been described before [21, 22]. Anyway, when the patients inherited both mutations from their parents, the presence of severe and/or persistent symptoms could be explained by combined effect of the two mutations. Our data confirm the possible occurrence of both major and minor form of the pathology, within the same family, as previously described in literature [15]. However, what is new is that the two brothers have different form of the disease, even if the compound heterozygous mutations in *GLRA1* are the same. It is described that parents with minor form can have children with major form or vice versa, as in our family, where mother shows a very mild form of the disease, but usually siblings tend to be affected by the same degree [11]. The presence of two clinical forms in two siblings could be explained as different phenotypic expressions of the same autosomal dominant gene. Both compound heterozygous patients and homozygous mutation carriers have been described in the literature for recessive forms of the disease [19, 23]. Dominant forms of hyperekplexia have been attributed to mutations within the pore-lining transmembrane segment (TM2) and adjacent regions, while recessive forms have been attributed to mutations within the other transmembrane segments (TM1 and TM3) [18, 24, 25]. The main outcome in our study is that these two known mutations in *GLRA1* have not been reported before as compound mutations, and that the p.R299X nonsense mutation detected in exon 7 of our patients, codifying TM3 domain, exhibited an autosomal dominant inheritance.

In conclusion, our data show that the same mutation in *GLRA1* could have different phenotypic expressions, suggesting an underling mechanism of variable expressivity, even in the same pedigree. Genetic testing of glycinergic neurotransmission-associated genes, including *GLRA1*, is a readily available tool either to confirm clinically suspected diagnosis of HPX or for family screening. Therefore, early recognition is helpful for prompt and appropriate treatment, to avoid unnecessary investigation and may lead to genetic preconception counseling.

## Abbreviations

CZP: Clonazepam
ECD: Extracellular domain
EEG: Electroencephalogram
GABA-A: γ-aminobutyric acid type A
HPX: Hyperekplexia
HRR: Exaggerated head-retraction reflex
TMD: Transmembrane domain
